# 170th anniversary of Graefe’s Archive for Clinical and Experimental Ophthalmology

**DOI:** 10.1007/s00417-024-06614-7

**Published:** 2024-08-30

**Authors:** Andrzej Grzybowski, Katarzyna Pawlikowska – Łagód, Martin Rohrbach

**Affiliations:** 1 Institute for Research in Ophthalmology, Foundation for Ophthalmology Development, Ul. Mickiewicza 24/3B, 60-836 Poznań, Poland; 2 Department of Humanities and Social Medicine, Medical University of Lublin, Ul. Chodźki 7, 20–093 Lublin, Poland; 3 Eye Hospital, Research Area „History of Ophthalmology”, Eberhard-Karls-University, Tübingen, Germany

**Keywords:** Graefe’s archive for clinical and experimental ophthalmology, Albrecht von graefe, Archive für augenheilkunde, Ophthalmology

## Abstract

**Abstract:**

The year 2024 marks the 170th anniversary of the journal Graefe’s Archive for Clinical and Experimental Ophthalmology. The journal is the first printed journal specializing in ophthalmology, founded by Albrecht von Graefe (1828–1870) in 1854. The reason behind creating the journal was to publish useful clinical information and develop discussion and debate among ophthalmologists and vision scientists. Thanks to its diligence and appropriate selection of published content, the journal has become widely read not only among the German scientific community, but also internationally. The aim of this review article is to present the activities of Graefe’s Archive for Clinical and Experimental Ophthalmology during the past 170 years.

****Key messages**:**

***What is known***

- Graefe's Archive for Clinical and Experimental Ophthalmology is the oldest worldwide ophthalmology journal that in 2024 celebrates its 170th anniversary.
- The journal was founded by Albrecht von Graefe, and after his death continued by other giants in ophthalmology, including Ferdinand von Arlt and Franciscus Cornelius Donders.

*****What is new***:**

- There were mostly male editors-in-chief, with Antonia Joussen as the first female editor-in-chief in the long journal's history.
- This article presents for the first time the complete list of all editors-in-chief in the 170 year long history of the journal.

## Introduction

In 2024, *Graefe’s Archive for Clinical and Experimental Ophthalmology*, the oldest ophthalmology journal in the world, celebrates its 170th anniversary. Founded by Albrecht von Graefe (1828–1870) in 1854 in Germany in the German language as a journal for German speakers. The journal, developed through the years with different names, survived two world wars, and numerous political changes throughout Germany and Europe. The purpose of founding the journal was to promote ophthalmic knowledge through the print media and to initiate discussion and debate among the ophthalmic community. Over the years, the journal expanded beyond the borders of Germany and became a renowned journal with an international reach [1, 2].

## Founding of Graefe’s archive for clinical and experimental ophthalmology

Ophthalmology gained its independence in the second half of the nineteenth century. At the same time, due to the industrial revolution with railroad development, people began to travel frequently not only between cities but also between countries, and this enabled better international exchange of knowledge, also in ophthalmology [3].

The exact beginning of modern ophthalmology is considered to date when the first ophthalmoscope was invented in 1851 by Hermann Helmholtz (1821–1894). In 1854, Albrecht von Graefe, who is considered the founder of modern ophthalmology, founded in Berlin the journal *Archiv für Ophthalmologie* and became its first editor-in-chief. In 1871, after Graefe’s death, the name of the journal was changed to *Albrecht von Graefes Archiv für Ophthalmologie*. This name lasted until 1965, at which time the name was changed to *Albrecht von Graefes Archiv für klinische und experimentelle Ophthalmologie.* Today, the journal is known by its English-language name: *Graefe’s Archive for Clinical and Experimental Ophthalmology* (Table 1). The journal has been publishing in English since 1981. [4–6]. The current publisher of the journal is Springer Nature (Table 2). Graefe's preface to the first volume from 1854 (in German) presents a "constitution" of “Graefe's Archive”:

“The need to have a separate organ in national literature dedicated to ophthalmology has long been felt by every specialist who is involved in the deeper study of the field mentioned. Given the current state of research, the works are too detailed to not have to be confined to the journals intended for medical sciences in general. […] We see the fog that has surrounded the best researchers in their insight for centuries disappearing under our eyes, and thanks to early knowledge, an unimagined field has been opened up for therapy, from which we can already bear beautiful fruit after just a few years. […] If, for the reasons given, the creation of an organ for ophthalmology is recognized as an urgent need, it may seem wonderful to some specialists that I, as an even younger worker in this field, have embarked on such an undertaking. In fact, I can assure you that it was not an overestimation of my own strength that brought me to this point, but simply the realization that the desired beginning had not been made by someone else.”

**Table 1:** Name of the journal by year

Year	Name of the magazine
1854—1870	Archiv für Ophthalmologie
1871—1964	Albrecht von Graefes Archiv für Ophthalmologie
1965—1981	Albrecht von Graefes Archiv für klinische und experimentelle Ophthalmologie Albrecht von Graefe’s Archive for Clinical and Experimental Ophthalmology
1981—1983	Graefe’s Archive for Clinical and Experimental Ophthalmology Albrecht von Graefes Archiv für klinische und experimentelle Ophthalmologie
1983 – till today	Graefe’s Archive for Clinical and Experimental Ophthalmology

**Table 2:** The journal’s publishers

Years	Publishers
1854—1855	P. Jeanrenaud, Berlin
1855—1886	Hermann Peters, Berlin
1887 – 1916	Wilhelm Engelmann, Leipzig
1917—today	Julius Springer Berlin, Heidelberg, and now Springer Nature

## Founder and editor-in-chief Albrecht von Graefe

**Fig. 1:**
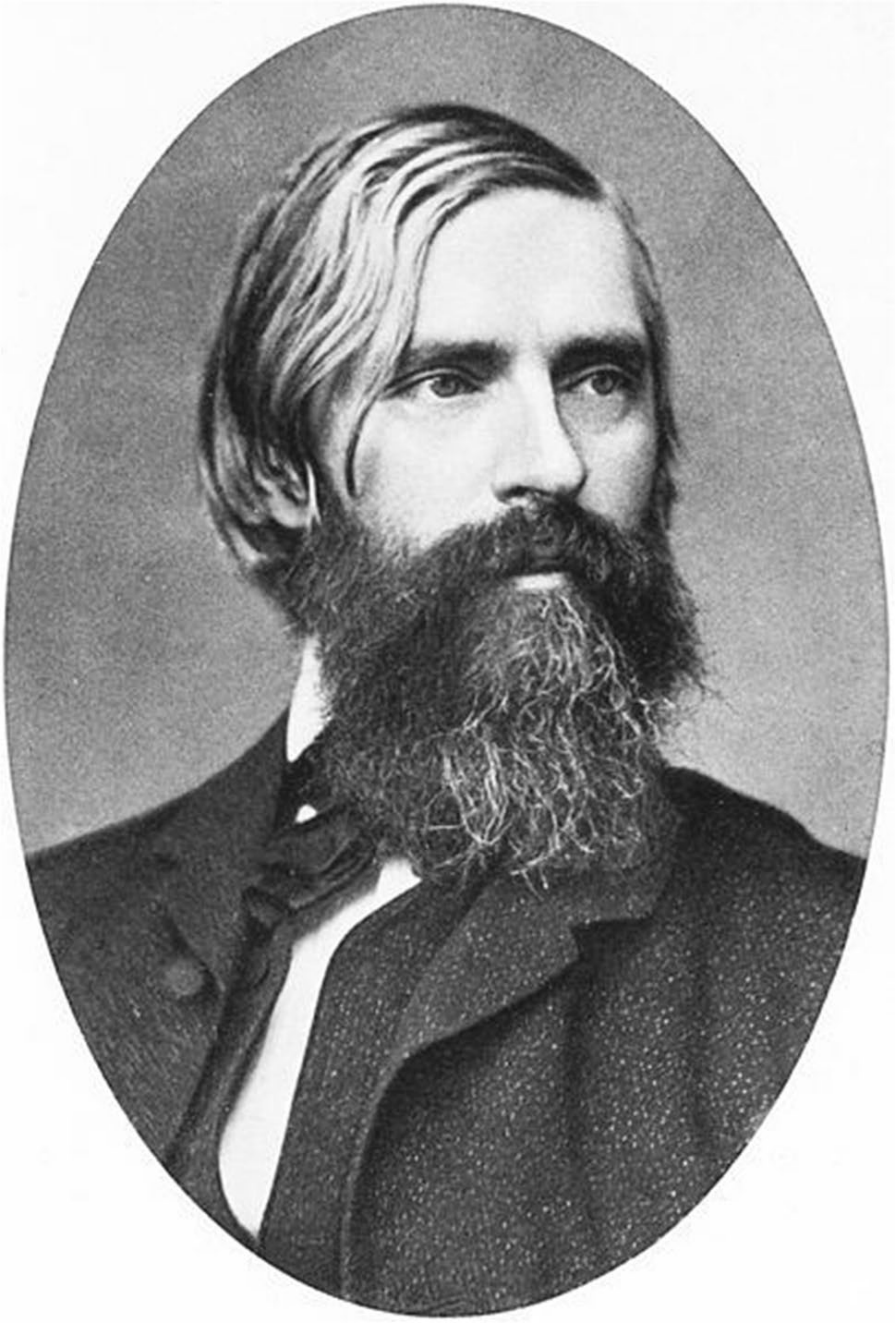
Friedrich Wilhelm Ernst Albrecht von Graefe (22.05.1828 – 20.07.1870). Source: https://commons.wikimedia.org/wiki/File:Albrecht_v_Graefe3.jpg

Albrecht (more precisely Friedrich Wilhelm Ernst Albrecht) von Graefe (Fig. 1) was born on May 22, 1828 in Finkenheerd, Brandenburg, and died on July 20, 1870 in Berlin. He was the son of Auguste von Alten (1797–1857) and Carl Ferdinand von Graefe (1787–1840). Graefe’s parents were wealthy and familiar with Austrian and Polish upper-class society. They were cosmopolitans. [7]. His father was born in Warsaw, where their family received nobility. He became a respected professor, surgeon and early ophthalmologist. He is considered the father of modern facial plastic surgery. He introduced many reconstructive operations, including cleft palate operation, plastic surgery of the nose. [8]. He held the position of director of German military hospitals during the Napoleonic wars and was director of the surgical clinic at the University of Berlin from 1810 to 1840. [9].

Albrecht received his elementary education from private teachers. After its completion, he attended the French gymnasium in Berlin. He graduated with honors at the age of 15. From 1843 to 1847 he studied medicine, where he also learned logic, philosophy, natural sciences and anatomy. His teachers included: Johannes Müller (1801–1858), leading physiologist of the nineteenth century, Johann Lukas Schönlein (1793–1864), remembered for his work on IgA vasculitis (Henoch–Schönlein purpura), Moritz Heinrich Romberg (1795–1873), neurologist who described the Romberg sign, and Rudolf Virchow (1821–1903), the famous pathologist (“Virchow’s trias”). In 1847, he received his doctorate on the basis of his thesis *“De bromo ejusque praeparatis.”* (“Of bromine and its preparations”). In the winter of 1847/48 he passed the state exam with a grade of “exemplary good”, and in 1848, he went to Prague to be trained under Carl Ferdinand von Arlt (1812–1887). Beginning his trip to Prague, von Graefe was undecided on which field of medicine he would pursue, but after observing Arlt’s ophthalmic skills, he made up his mind that ophthalmology would be his field.

Later, he left Prague for Paris, where he learned experimental physiology, including extraocular muscles and optic nerve function with Claude Bernard (1813–1878), major European physiologist of that time. In Paris, he was also a regular visitor to the ophthalmology clinic of Jules Sichel (1802–1868), where he also met Louis-Auguste Desmarres (1810–1882). He then studied in Vienna in years 1849–1850 under Christopher Friedrich Jäger Ritter von Jaxtthal (1784–1871) and Eduard Jaeger Ritter von Jaxtthal (1818–1884), which gave him extensive opportunities to develop ophthalmic practice. He then went to London to Moorfields Eye Hospital, where he met William Bowman (1816–1892), George Critchett (1817–1882). He also met in London Franciscus Cornelis Donders (1818–1889), with whom Graefe became friends and began collaborating in research [7, 10].

In 1850, Graefe returned to Berlin richer in medical and scientific knowledge. He opened his own clinic, which was based on observations of French clinics. At the clinic, Graefe was involved not only in the clinical work, but also in teaching, and research [11]. In the future years, when he became internationally recognized, representatives of science and medicine from all over the world visited the clinic. In 1852, he received his habilitation based on his work entitled “*Über die Wirkung der Augenmuskeln”(About the action of the eye muscles).* In 1857, he founded the German Ophthalmological Society, which is the oldest scientific society in the world. In the same year he was appointed associate professor of ophthalmology in Berlin – the first German professor of eye diseases, while in 1866 he was appointed full professor [10]. He also became a unique figure in the international arena, presiding over and dominating the entire Third International Congress of Ophthalmology, held in Paris in 1867 [12–14].

Von Graefe tended to study ophthalmology in all its glory. However, there were certain areas in which he took a special interest. His student Julius Hirschberg (1843–1925) introduced them as follows:

- The first period until 1857 – he was interested in conjunctival diseases, sensory physiology and strabismus. In 1851, he was one of the first to use the ophthalmoscope invented by Hermann von Helmholtz (1821–1894);
- The second period until 1863 – he dealt with glaucoma and introduced peripheral iridectomy for the treatment of glaucoma in 1857. The introduction of iridectomy is considered one of Graefe’s greatest ophthalmological discoveries;
- The third period lasted until his death – Graefe was involved in glaucoma and cataract removal [15].

At the age of 33, he contracted acute pulmonary tuberculosis, as a result of which he died in 1870 at just 42 years old. He left behind his wife Anna Knuth and five children.

## Editors-in-chief

**Fig. 2:**
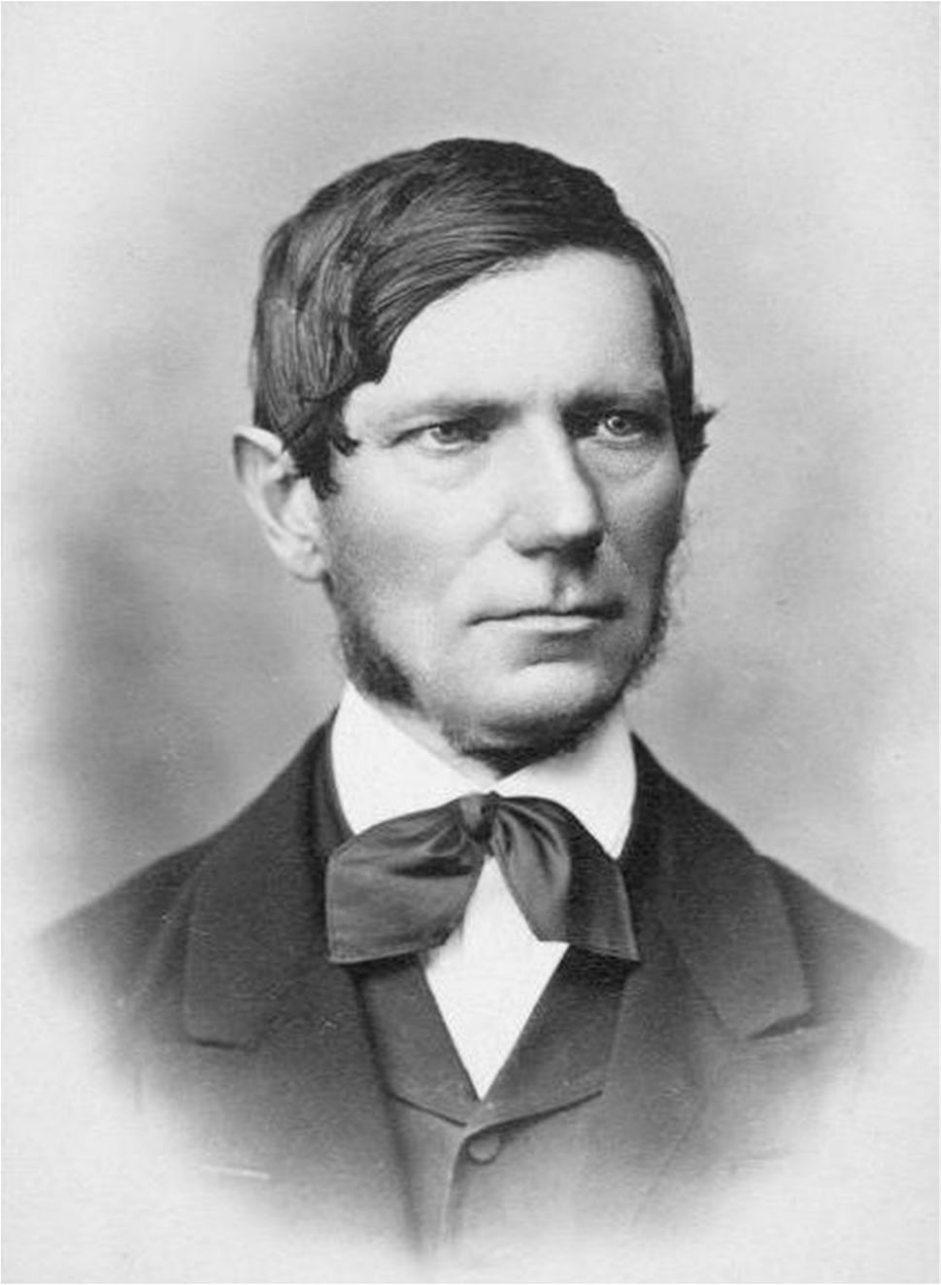
Ferdinand von Arlt (1812–1887). Source: https://en.wikipedia.org/wiki/Carl_Ferdinand_von_Arlt#/media/File:Luckhardt_-_Carl_Ferdinand_von_Arlt_(%C3%96NB_8075127).jpg

**Fig. 3:**
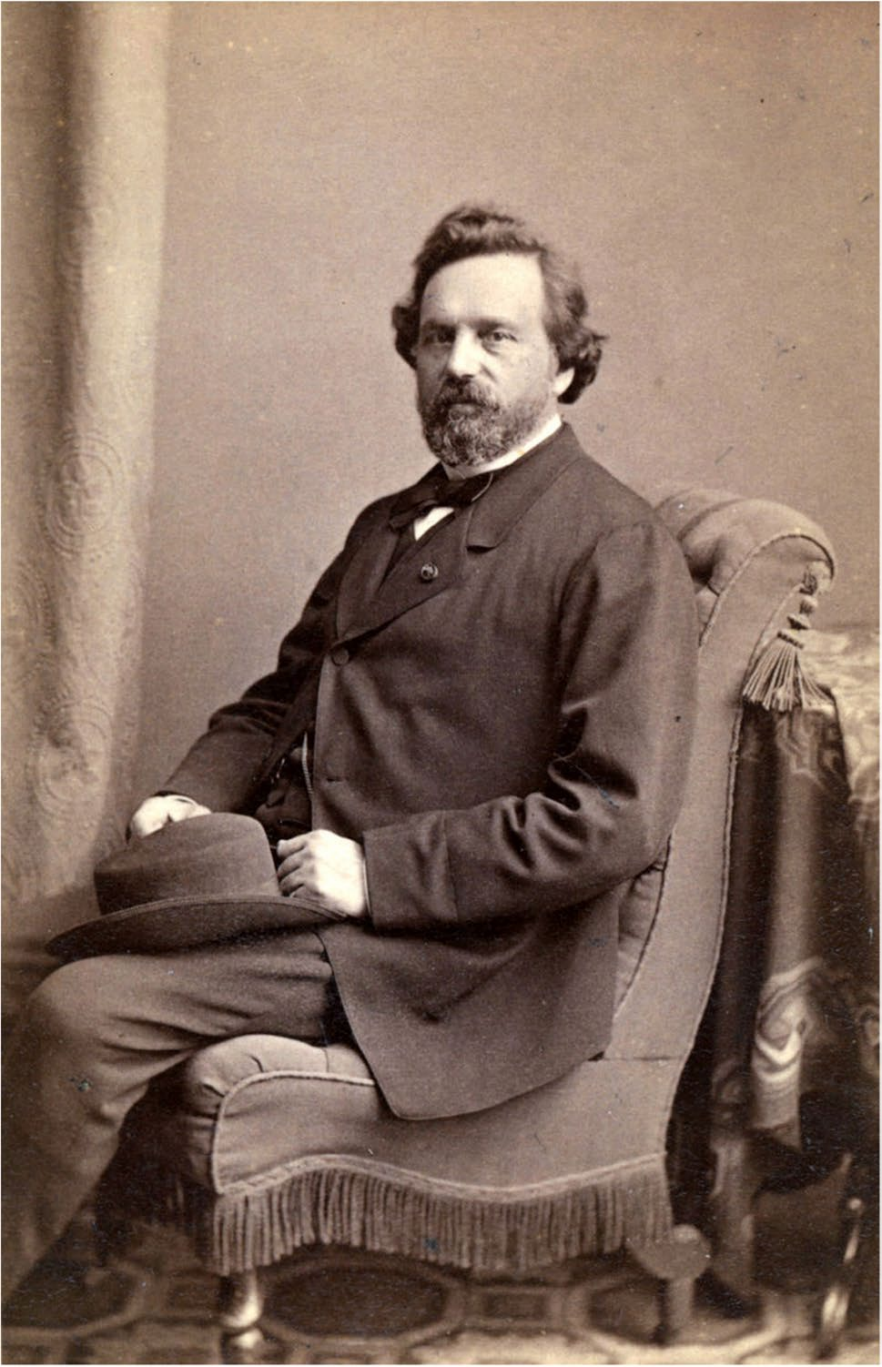
Franciscus Cornelius Donders (1818–1889). Source: https://en.wikipedia.org/wiki/Franciscus_Donders#/media/File:Donders,_Franciscus_Cornelis_(1818_-_1889).jpg

After the death of Albrecht von Graefe, Ferdinand von Arlt (Fig. 2) and Franciscus Cornelius Donders (Fig. 3) became editors-in-chief. Ferdinand von Arlt (1812–1887) was an Austrian ophthalmologist and surgeon. Among many of his contributions, he was the first to show that myopia is usually caused by overextension of the sagittal axis of the eye [16]. Franciscus Cornelis Donders was a Dutch ophthalmologist, mainly known for his research on the accommodation of the eye [17]. Theodor Leber (1840–1917) was a prominent German ophthalmologist, known mainly for his research on hereditary retinal diseases. In 1869, he described the syndrome now known as congenital amaurosis of Leber, and in 1871, Leber’s hereditary optic neuropathy [18]. Ernst Fuchs (1851–1930) was a well-known Austrian ophthalmologist and pathologist, who worked on the pathology of the eye. His work entitled "Textbook of Ophthalmology" became known as a masterpiece of ophthalmology in the nineteenth century. In ocular oncology, Fuchs was the first to introduce “sarcom des uvealtractus” (uveal sarcoma), known today as uveal melanoma [19]. David Glendenning Cogan (1908–1993) was an American ophthalmologist and neurologist, best known for his contributions to the field of neuro-ophthalmology, “Cogan’s corneal dystrophy” and “Cogan’s syndrome” [20]. Gerhard Rudolf Edmund Meyer-Schwickerath (1920–1992) is considered one of the pioneers of light and laser therapy in ophthalmology. His works allowed many patients with retinal diseases to be treated, changing the way ophthalmologists deal with diabetic retinopathy and other eye diseases [21]. Klaus Heimann (1935–1999) was a German pioneer of vitreo-retinal surgery [22].

The editorship from 1948 is also noteworthy. Ernst Engelking, the best-known Nazi critic among ophthalmologists, and Carl Wessely, the expelled full professor in Munich because he was Jewish, were appointed. Walther Löhlein also worked as editor from 1948. Löhlein led the DOG (Die Deutsche Ophthalmologische Gesellschaft—German Society of Ophthalmology) through the Nazi era and was Hitler's ophthalmologist. While in the recent past the editors often did not serve for very long, this was usually different in the past. The activity often lasted for decades and usually only ended with death. Terms of office of exemplary editors (years) are as following:

- August Wagenmann (1863 – 1955) – 55 years,
- Theodor Leber (1840—1917) – 46 years,
- Hubert Sattler (1844 – 1928) – 42 years,
- Franciscus Cornelis Donders (1818—1889) – 34 years,
- Carl Ferdinand von Arlt (1812—1887) – 31 years,
- Eugen Schreck (1911–1993) – 28 years,
- Ernst Engelking (1886 – 1975) – 26 years,
- Walther Löhlein (1882 – 1954) – 26 years,
- Albrecht von Graefe (1828 – 1870) – 16 years,
- Antonia Joussen – 13 years,
- David Glendenning Cogan (1908 – 1993) – 9 years.

It is worthy highlighting that there were mostly men editors, and Antonia Joussen is the first woman editor-in-chief in the 170th years of the journal history.

Table 3 presents editors-in-chief of the journal.

**Table 3:** Editors-in-chief of the journal

1854	Albrecht von Graefe (1828 – 1870)
1855—1870	Carl Ferdinand von Arlt (1812–1887) Franciscus Cornelis Donders (1818–1889) Albrecht von Graefe (1828 – 1870)
1871—1886	Carl Ferdinand von Arlt (1812–1887) Franciscus Cornelis Donders (1818–1889) Theodor Leber (1840–1917)
1887	Franciscus Cornelis Donders (1818–1889) Theodor Leber (1840–1917)
1888—1889	Franciscus Cornelis Donders (1818–1889) Theodor Leber (1840–1917) Hubert Sattler (1844 – 1928)
1889	Theodor Leber (1840–1917) Hubert Sattler (1844 – 1928)
1890—1899	Theodor Leber (1840–1917) Hubert Sattler (1844 – 1928) Hermann Snellen (1834–1908)
1900—1907	Theodor Leber (1840—1917) Hubert Sattler (1844 – 1928) Hermann Snellen (1834 – 1908)
1908—1917	Ernst Fuchs (1851–1930) Theodor Leber (1840—1917) Hubert Sattler (1844 – 1928) August Wagenmann (1863 – 1955)
Editing:	Theodor Leber (1840—1917) August Wagenmann (1863 – 1955)
1917	Ernst Fuchs (1851–1930) Hubert Sattler (1844 – 1928) August Wagenmann (1863 – 1955)
1917—1928	Ernst Fuchs (1851–1930) Eugen von Hippel (1867 – 1939) Hubert Sattler (1844 – 1928) August Wagenmann (1863 – 1955)
1928–1930	Alfred Bielschowsky (1871 – 1940) Arthur Brückner (1877–1975) Ernst Fuchs (1851–1930) Eugen von Hippel (1867 – 1939) Karl Lindner (1883—1961) Walther Löhlein (1882 – 1954) Hubert Sattler (1844 – 1928) Franz Schieck (1871–1946) Erich Seidel (1882 – 1946) Alfred Vogt (1879 – 1943) August Wagenmann (1863 – 1955)
Editing:	August Wagenmann (1863 – 1955)
1930—1934	Alfred Bielschowsky (1871 – 1940) Arthur Brückner (1877–1975) Eugen von Hippel (1867 – 1939) Karl Lindner (1883—1961) Walther Löhlein (1882 – 1954) Franz Schieck (1871–1946) Erich Seidel (1882 – 1946) Alfred Vogt (1879 – 1943) August Wagenmann (1863 – 1955)
1935–1938	Arthur Brückner (1877–1975) Eugen von Hippel (1867 – 1939) Karl Lindner (1883—1961) Walther Löhlein (1882 – 1954) Franz Schieck (1871–1946) Erich Seidel (1882 – 1946) Alfred Vogt (1879 – 1943) August Wagenmann (1863 – 1955)
1938—1943	Ernst Hertel (1870–1943) Walther Löhlein (1882–1954) August Wagenmann (1863 – 1955)
1944	Walther Löhlein (1882 – 1954) August Wagenmann (1863 – 1955)
1948—1952	Ernst Engelking (1886 -1975) Walther Löhlein (1882 – 1954) Oswald Marchesani (1900 – 1952) August Wagenmann (1863 – 1955) Karl Wessely (1874 – 1953)
1952—1953	Ernst Engelking (1886 – 1975) Walther Löhlein (1882 – 1954) Hans – Karl Müller (1899 – 1977) August Wagenmann (1863 – 1955) Karl Wessely (1874 – 1953)
1953—1954	Ernst Engelking (1886 – 1975) Walther Löhlein (1882 – 1954) Hans – Karl Müller (1899 – 1977) August Wagenmann (1863 – 1955)
1954—1955	Ernst Engelking (1886 – 1975) Hans – Karl Müller (1899 – 1977) Eugen Schreck (1911–1993) August Wagenmann (1863 – 1955)
1955—1961	Ernst Engelking (1886 – 1975) Hans – Karl Müller (1899 – 1977) Eugen Schreck (1911–1993)
1961—1964	Josef Böck (1901 – 1985) Ernst Engelking (1886 – 1975) Hans-Karl Müller (1899 – 1977) Eugen Schreck (1911–1993)
1965—1970	Josef Böck (1901 – 1985) Ernst Engelking (1886 – 1975) Hans – Karl Müller (1899 – 1977) Eugen Schreck (1911–1993) Eugen Schreck (1911–1993)
1970	Josef Böck (1901 – 1985) Ernst Engelking (1886 – 1975) Hans – Karl Müller (1899 – 1977) Friedrich Rintelen (1906–1991) Eugen Schreck (1911–1993) Charles Thomas (1906–1987)
1971	Josef Böck (1901 – 1985) Robert Arnold Crone (1918 – 2012) Ernst Engelking (1886 – 1975) Hans – Karl Müller (1899 – 1977) Friedrich Rintelen (1906–1991) Eugen Schreck (1911–1993) Charles Thomas (1906–1987) Manuel Straub (1894 – 1961)
1972—1973	Josef Böck (1901 – 1985) David Glendenning Cogan (1908 – 1993) Robert Arnold Crone (1918 – 2012) Ernst Engelking (1886 – 1975) Hans – Karl Müller (1899 – 1977) Friedrich Rintelen (1906–1991) Eugen Schreck (1911–1993) Wolfgang Straub (1920–1993) Charles Thomas (1906–1987)
1974	Frederick C. Blodi (1917 – 1996) Josef Böck (1901 – 1985) David Glendenning Cogan (1908 – 1993) Robert Arnold Crone (1918 – 2012) Ernst Engelking (1886 – 1975) Gerhard Meyer-Schwickerath (1920 – 1992) Hans – Karl Müller (1899 – 1977) Friedrich Rintelen (1906–1991) Eugen Schreck (1911–1993) Wolfgang Straub (1920–1993) Charles Thomas (1906–1987)
1975	Frederick C. Blodi (1917–1996) Josef Böck (1901 – 1985) David Glendenning Cogan (1908 – 1993) Robert Arnold Crone (1918 – 2012) Gerhard Meyer-Schwickerath (1920 – 1992) Hans – Karl Müller (1899 – 1977) Friedrich Rintelen (1906–1991) Eugen Schreck (1911–1993) Wolfgang Straub (1920–1993) Charles Thomas (1906–1987)
1976—1979	Gerhard Meyer-Schwickerath (1920 – 1992) Bradley Straatsma Eugen Schreck (1911–1993) Wolfgang Straub (1920–1993)
1979—1980	David Glendenning Cogan (1908 – 1993) Gerhard Meyer-Schwickerath (1920 – 1992) Eugen Schreck (1911–1993) Bradley Straatsma Wolfgang Straub (1920–1993)
1981	Gerhard Meyer-Schwickerath (1920 – 1992) Eugen Schreck (1911–1993) Eugen Schreck (1911–1993) Bradley Straatsma Wolfgang Straub (1920–1993)
1982	Alan Charles Bird Gerhard Meyer-Schwickerath (1920 – 1992) Eugen Schreck (1911–1993) Bradley Straatsma Wolfgang Straub (1920–1993)
1983—1987	Alan Charles Bird Robert Machemer (1933 – 2009), Gerhard Meyer-Schwickerath (1920 – 1992) Manfred Spitznas Bradley Straatsma
1988—1990	Stephen Michael Drance (1925 – 2020) William Lee Robert Machemer (1933 – 2009) Manfred Spitznas Rainer Sundmacher
1991	Klaus Heimann (1935 – 1999) Günther Krieglstein William Le Robert Machemer (1933 – 2009) Rainer Sundmacher
1992—1996	Klaus Heimann (1935 – 1999) George W. Blankenship (1940–2020) Günther Krieglstein William Lee Robert Machemer (1933 – 2009)
1997–1998	Klaus Heimann (1935 – 1999) William Lee Günther Krieglstein Robert Machemer (1933 – 2009)
1999–2002	William Lee Günther Krieglstein
2002–2003	Günther Krieglstein
2003—2010	Bernd Kirchhof
2011—2018	Antonia Joussen David Wong
2019—to date	Antonia Joussen Srinivas Sadda Taiji Sakamoto

## Development of the journal

Graefe’s Archive for Clinical and Experimental Ophthalmology was published as a yearbook during its first years (until 1862). From 1863 onward, two to three volumes were published annually, except for 1870 when only one issue was published. In turn, from 1876 onward, three to four issues per year were published. In 1953, six issues were published. And from that year the publishing trend reached 4 to 6 issues per year. Since 1993, Graefe’s Archive for Clinical and Experimental Ophthalmology has become a monthly journal [23].

Between 1854 and 1869, editor-in-chief Albrecht von Graefe alone filled some 2,500 pages of the journal, largely with his case reports, to which he attached particular importance. In the first issue, 400 of the 480 pages were written by him.

Some first descriptions appeared in the early issues of Archive were as follows:

- 1855 – the excavation of the optic disc in glaucoma [24],
- 1857 – iridectomy for the treatment of glaucoma [25],
- 1859 – occlusion of the central retinal artery [26],
- 1860 – optic neuritis in “diseases of the brain” [27],
- 1866 – swelling of the optic disc with increased intracranial pressure [15, 28].

Initially, the journal was aimed at German readers. However, over time, as a result of its increased prestige, it has become an international journal. Today, the journal is published by the editors of Springer Nature and is an international journal that publishes original clinical studies and relevant experimental research in ophthalmology. It is the official journal of the German Ophthalmology Society, and Italian Society of Vitreoretinal Surgeons.

In 1854, when the journal was founded, 8 articles were published. Until 1988, the number of articles published per year oscillated between 16 and 120. Only since 1989 has there been an annual increase in the number of published articles. The highest number, 474 articles to be exact, was published in 2021.

Readers come from every corner of the world. The most articles in the journal from 2019 to 2022 were published by authors coming from the US, China and Germany. However, among the organizations that published the most articles in the journal during the indicated time period were Tel Aviv University (43), University of California (40), and University of Cologne (38).

Table 4 shows the 10 most cited papers to date.

**Table 4:** Top 10 most cited papers in Graefe's Archive for Clinical and Experimental Ophthalmology

	Title	PubYear	Volume	Issue	Times cited
1.	Haigis W, Lege B, Miller N, Schneider B. Comparison of immersion ultrasound biometry and partial coherence interferometry for intraocular lens calculation according to Haigis	2000	238	9	597
2.	Bennett AG, Rudnicka AR, Edgar DF. Improvements on Littmann's method of determining the size of retinal features by fundus photography	1994	232	6	525
3.	Lange C, Feltgen N, Junker B, Schulze-Bonsel K, Bach M Resolving the clinical acuity categories “hand motion” and “counting fingers” using the Freiburg Visual Acuity Test (FrACT)	2008	247	1	440
4.	Wu L, Martínez-Castellanos MA, Quiroz-Mercado H, Arevalo JF, Berrocal MH, Farah ME, Maia M, Roca JA, Rodriguez FJ; Pan American Collaborative Retina Group (PACORES) Twelve-month safety of intravitreal injections of bevacizumab (Avastin®): results of the Pan-American Collaborative Retina Study Group (PACORES)	2007	246	1	415
5.	Sebag J Anomalous posterior vitreous detachment: a unifying concept in vitreo-retinal disease	2004	242	8	397
6.	Schirmer O Studien zur Physiologie und Pathologie der Tränenabsonderung und Tränenabfuhr	1903	56	2	373
7.	Lai THT, Tang EWH, Chau SKY, Fung KSC, Li KKW Stepping up infection control measures in ophthalmology during the novel coronavirus outbreak: an experience from Hong Kong	2020	258	5	303
8.	Wang X, Jiang C, Ko T, Kong X, Yu X, Min W, Shi G, Sun X Correlation between optic disc perfusion and glaucomatous severity in patients with open-angle glaucoma: an optical coherence tomography angiography study	2015	253	9	303
9.	Algvere PV, Berglin L, Gouras P, Sheng Y Transplantation of fetal retinal pigment epithelium in age-related macular degeneration with subfoveal neovascularization	1994	232	12	296
10.	Machemer R, Steinhorst UH Retinal separation, retinotomy, and macular relocation II. A surgical approach for age-related macular degeneration?	1993	231	11	295

**Fig. 4:**
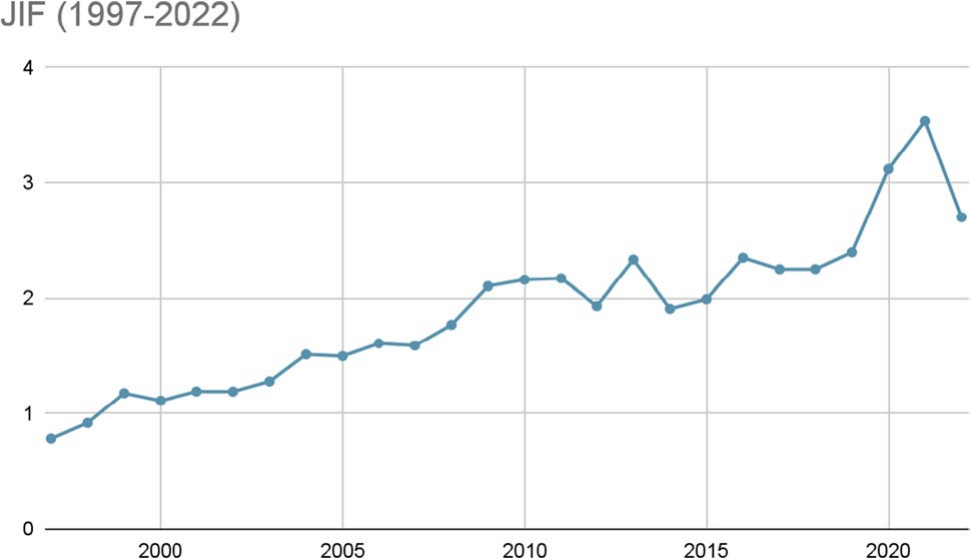
Graefe's Archive for Clinical and Experimental Ophthalmology journal IF

The journal *Graefe’s Archive for Clinical and Experimental Ophthalmology* is ranked 28 (out of 62) on the list of ophthalmology journals listed in the Journal Citation Reports database relative to its Impact Factor index for 2022. The Fig. 4 shows the Impact Factor index for *Graefe’s Archive for Clinical and Experimental Ophthalmology*. The total number of citations (JCR) was 11,731, and the journal citation index (JCI) was 1.05. According to the journal citation index (JCI) ranking, the journal was ranked 24 out of 95 [29].

## Conclusions

The history of Graefe’s Archives for Clinical and Experimental Ophthalmology is not only about the journal itself, but also about the history and the development of ophthalmology as a field of medicine and science. Representing the first ophthalmic journal, it developed the foundations of modern ophthalmology. The journal’s influence is lasting and relevant to modern medical practice and research.
